# Fibroblasts in fibrosis: novel roles and mediators

**DOI:** 10.3389/fphar.2014.00123

**Published:** 2014-05-27

**Authors:** Ryan T. Kendall, Carol A. Feghali-Bostwick

**Affiliations:** Division of Rheumatology and Immunology, Department of Medicine, Medical University of South CarolinaCharleston, SC, USA

**Keywords:** fibroblast, myofibroblast, fibrosis, scleroderma, idiopathic pulmonary fibrosis, extracellular matrix, endostatin

## Abstract

Fibroblasts are the most common cell type of the connective tissues found throughout the body and the principal source of the extensive extracellular matrix (ECM) characteristic of these tissues. They are also the central mediators of the pathological fibrotic accumulation of ECM and the cellular proliferation and differentiation that occurs in response to prolonged tissue injury and chronic inflammation. The transformation of the fibroblast cell lineage involves classical developmental signaling programs and includes a surprisingly diverse range of precursor cell types—most notably, myofibroblasts that are the apex of the fibrotic phenotype. Myofibroblasts display exaggerated ECM production; constitutively secrete and are hypersensitive to chemical signals such as cytokines, chemokines, and growth factors; and are endowed with a contractile apparatus allowing them to manipulate the ECM fibers physically to close open wounds. In addition to ECM production, fibroblasts have multiple concomitant biological roles, such as in wound healing, inflammation, and angiogenesis, which are each interwoven with the process of fibrosis. We now recognize many common fibroblast-related features across various physiological and pathological protracted processes. Indeed, a new appreciation has emerged for the role of non-cancerous fibroblast interactions with tumors in cancer progression. Although the predominant current clinical treatments of fibrosis involve non-specific immunosuppressive and anti-proliferative drugs, a variety of potential therapies under investigation specifically target fibroblast biology.

## Introduction

Fibroblasts are the workhorse of the most important tissue that holds the human body together—connective tissue. Connective tissue joins and supports all other tissues, including the parenchymal tissues of organs. This connective tissue is made of fibroblasts widely-spaced in a vast extracellular matrix (ECM) of fibrous proteins and gelatinous ground substance. Fibroblasts produce the ECM's structural proteins (e.g., fibrous collagen and elastin), adhesive proteins (e.g., laminin and fibronectin), and ground substance (e.g., glycosaminoglycans, such as hyaluronan and glycoproteins). However, fibroblasts also play various additional roles beyond ECM production. For example, fibroblasts serve pivotal roles in ECM maintenance and reabsorption, wound healing, inflammation, angiogenesis, cancer progression, and in physiological as well as pathological tissue fibrosis. Ancillary to these various biological roles, fibroblasts produce and respond to a broad array of paracrine and autocrine signals, such as cytokines and growth factors. Targeting these ancillary signaling events is the main strategy underlying multiple lines of research for a new generation of treatments for fibroblast-related disorders.

Fibroblasts are mesenchymal cells derived from the embryonic mesoderm tissue, and they are not terminally differentiated. They can be activated by a variety of chemical signals that promote proliferation and cellular differentiation to form myofibroblasts with an up-regulated rate of matrix production. Fibroblast activation plays a vital role in wound healing however, in some cases and for reasons that remain to be fully elucidated, their activation becomes uncontrolled, producing a pathological fibrotic response that promotes multiple diseases and affects a variety of organs. Indeed, fibrosis plays a significant contributory role in most cases of organ failure. Examples are wide ranging: systemic sclerosis (SSc); idiopathic pulmonary fibrosis (IPF); liver cirrhosis; kidney fibrosis; and the cardiac fibrosis observed in cardiac hypertrophy resulting in heart failure. The essential role of fibroblasts in lung fibrosis was validated using lineage-specific deletion of the type II TGFβ receptor (Hoyles et al., 2011).

Here we review a variety of fibroblast functions that illustrate a central role for fibroblasts in the pathology of fibrosis. We review ECM production in relation to fibrosis, some examples of critical chemical signaling, myofibroblast differentiation, the role of fibroblasts in stromal-cancer interactions, and potential clinical therapies targeting fibroblasts.

## Fibroblast role in matrix production and maintenance

Fibroblasts' most well-known biological role is the production of the rich ECM of connective tissues. Fibroblasts produce and secrete all components of the ECM, including the structural proteins, adhesive proteins, and a space-filling ground substance composed of glycosaminoglycans and proteoglycans.

### Structural proteins

Fibroblasts produce an interconnecting meshwork of extracellular protein fibers and connector proteins that provide structure to tissue. Reciprocally, these proteins can promote the differentiation of profibrotic myofibroblasts through positive feedback regulation during fibrosis (Blaauboer et al., 2013). The variety of ECM structures serve roles as diverse as forming lamina that delineate borders separating distinct cell types with different functions, to serving as interstitial regions that connect cells of a common type with a common biological function. Fibroblasts provide these specialized ECMs in different tissues by expressing and secreting a robust and variable repertoire of structural proteins with distinct properties. For example, the fibril rigidity provided by collagen type I, the most abundant protein in mammals, is due to its rope-shaped, triple-stranded helical tertiary protein structure that reinforces its tensile strength, preventing overstretching. In contrast, elastin proteins form highly crosslinked, yet unstructured, elastic networks that allow for expansive stretching without breaking. Tissues requiring differing degrees of rigidity vs. flexibility, such as skin and lung, differ in the relative expression of collagen subtype and elastin proteins. Moreover, the pathological histology of fibrosis includes an increase in the relative balance of collagen (Gilbane et al., 2013; McKleroy et al., 2013). A large number of genes that express different collagen subtypes also provide unique matrix properties. Fibroblast-mediated formation of basement membrane (composed of a layer of basal lamina and a layer of reticular lamina) serve as structural scaffolds critical for tissue regeneration in wound healing; cell barriers segregating epithelial from endothelial tissues; barriers preventing malignant escape or invasion of cancerous cells; filtration devices found in the glomerular filtration of blood in the kidney; and filtration of the alveoli-capillary interface in the lung. Basement membrane ECM is composed largely of collagen type IV, and in this environment, the presence of collagen type I promotes the up-regulation of TGFβ and epithelial-to-mesenchymal transdifferentiation (EMT), producing, for example, fibroblasts with a profibrotic phenotype in kidney fibrosis (Zeisberg et al., 2001).

### Adhesive proteins

Adhesive ECM proteins such as fibronectin and laminin form the connection between cells and the ECM. Fibronectins bind to ECM fibers and contain RGD domains required for interacting with the integrin transmembrane cell adhesion proteins. It is ironic that fibronectin is essential for collagen assembly into ECM, yet a conditional knock out of fibronectin expression in the liver was found to increase collagen production, TGFβ signaling, and hepatic fibrosis (Kawelke et al., 2011). Therefore, locally produced fibronectin in ECM also regulates the amount of active TGFβ and serves to protect tissue from TGFβ hyperstimulation (Kawelke et al., 2011). Laminins also interact with integrin receptors to regulate cell attachment to the basal lamina. In fact, laminin has been proposed as a biomarker for hepatic fibrosis (Santos et al., 2005).

### Ground substance

The ground substance of ECM is a hydrated gel of proteoglycans that is interspersed among the structural proteins. The ground substance forms a final pathway for nutrient flow beyond the reach of blood vessel transport into tissues as well as a pathway for intercellular communication. This cell-free medium forms an avenue for cell migration of immune cells, fibroblasts, and myofibroblasts. It is also an essential avenue for endothelial cell migration during angiogenesis. The proteoglycans of ground substance are proteins that are posttranslationally modified by the addition of unbranched polysaccharides called glycosaminoglycans (GAGs), or descriptively as mucopolysaccharides. These GAGs are composed of repeating disaccharide units that contain an amino sugar of either N-acetylglucosamine or N-acetylgalactosamine and are often sulphated. The family of GAG posttranslational modifications found in ECM includes hyaluronan (non-sulfated), heparin and heparan sulfate, chondroitin and dermatan sulfate, and keratan sulfate.

Proteoglycans directly interact with many chemical signals, such as growth factors and TGFβ. Through these interactions, the ECM proteoglycans can regulate chemical signaling by either inhibiting or augmenting a signal's responsiveness. The proteoglycan tenascin-C (TN-C) interacts with ECM (Chung et al., 1995), cell surface receptors (Schnapp et al., 1995; Midwood et al., 2009; De Laporte et al., 2013), and is secreted in response to TGFβ stimulation (Pearson et al., 1988). TN-C is highly expressed during embryonic development, chronic inflammation, and fibrosis. Our group has characterized the role of insulin-like growth factor binding protein (IGFBP)-3, which is overexpressed in fibrotic skin and lungs, in mediating the induction of TN-C by TGFβ (Brissett et al., 2012). Exposure of lung fibroblasts *in vitro* to IGFBP-3 directly induces TN-C production and secretion. Further, SSc patients with pulmonary fibrosis have significantly higher levels of circulating TN-C compared with SSc patients without pulmonary fibrosis (Brissett et al., 2012). Another ECM proteoglycan, decorin, antagonizes TGFβ activity (Yamaguchi et al., 1990). Decorin is a small proteoglycan that contains a single GAG chain of either chondroitin sulfate or dermatan sulfate. Decorin directly interacts with TGFβ and inhibits its profibrotic biological activity. Recombinant expression of decorin in the lung airways of mice inhibits bleomycin-induced pulmonary fibrosis (Kolb et al., 2001). In addition to the secreted proteoglycans of the ECM, there are also a number of cell membrane proteoglycans that can interact with chemical signals and function as co-receptors. An important example is the syndecans. Syndecan 2 (SDC2) is over-expressed during fibrosis and is induced in fibroblasts in response to both TGFβ and IGFBP-3 (Ruiz et al., 2012). Similar to the above examples, proteoglycans can also interact with membrane-bound and secreted proteases such as MMP-7 (Yu and Woessner, 2000) as well as secreted protease inhibitors such as TIMP-3 (Yu et al., 2000), regulating their biological activities in signaling and ECM processing.

### ECM maintenance and reabsorption

In addition to extracellular matrix production, fibroblasts are also responsible for its maintenance and reabsorption. Unlike bone, where a specialized cell type, osteoblasts, produces bone (a highly mineralized extracellular matrix) and a distinct cell type, osteoclasts, reabsorbs bone, there are no known fibroblast cells that specialize in matrix reabsorption. As far as we know, the same fibroblasts that produce ECM are responsible for matrix maintenance and degradation. As such, these cells have important roles in resolving pathological fibrosis. Collagen maturation is controlled by the enzyme lysyl oxidase (Lox), which is produced by fibroblasts, cross-links collagen fibers, and thereby strengthens ECM. Interestingly, inhibiting LOX-mediated collagen cross-linking antagonizes both fibrosis and tumor metastasis (Cox et al., 2013). Collagen turnover (catabolism) is regulated by a multitude of secreted extracellular proteases. Fibroblasts produce both matrix degrading enzymes (such as metalloproteinases, aka MMPs) as well as their inhibitors (the tissue inhibitors of metalloproteinases, aka TIMPs). Curiously, MMP expression in IPF is increased, and experiments with various MMP knockout mice demonstrate protection from bleomcyin-induced lung fibrosis (McKleroy et al., 2013).

## Concomitant and interwoven biological roles of fibroblasts

### Wound healing

Fibroblasts have a pivotal role in wound healing in response to tissue injury. First and foremost, fibroblasts respond to wound healing by proliferating and by chemotaxing to the sites of tissue injury to rebuild the ECM as a scaffold for tissue regeneration. Fibroblast to myofibroblast transitioning enables the contraction of the matrix to seal an open wound in the event of the loss of tissue (Gabbiani, 2003; Midwood et al., 2004). Fibroblasts also play a role in blood clotting, such as in the production of urokinase plasminogen activators (PAs) and their inhibitors (PAIs). Fibroblasts express the protease activated receptor PAR1 that enables fibroblast responsiveness to activated thrombin. PAR1 receptor expression is upregulated in IPF (Howell et al., 2005) and in lung tissue of SSc patients (Bogatkevich et al., 2005). Further, PAR1 knockout mice resist bleomycin-induced lung fibrosis (Howell et al., 2005). PAR1 receptors have well documented roles in the process of fibrosis, and PAR1 receptor antagonists as well as thrombin inhibitors could be beneficial for treating SSc and IPF (Atanelishvili et al., 2014).

### Inflammation

Fibroblasts serve roles in inflammation and immune cell recruitment to sites of tissue injury. Furthermore, fibroblasts produce and are responsive to many inflammatory cytokines. Fibroblasts are responsive to cytokines such as TGFβ1, IL-1β, interleukin-6 (IL-6), IL-13, IL-33 (Feghali and Wright, 1997; Scotton and Chambers, 2007; Savinko et al., 2012), as well as prostaglandins (Keerthisingam et al., 2001; Stratton et al., 2002; Maher et al., 2010) and leukotrienes (Mensing and Czarnetzki, 1984; Chibana et al., 2003; Eap et al., 2012). Fibroblasts are stimulated chemically by inflammatory agents to differentiate into myofibroblasts that have a greatly up-regulated rate of matrix production (discussed in more detail below). In turn, fibroblasts produce and secrete cytokines such as TGFβ1, IL-1β, IL-33, CXC, and CC chemokines (Feghali and Wright, 1997; Gharaee-Kermani et al., 2003, 2012; Scotton and Chambers, 2007), as well as reactive oxygen species (Amara et al., 2010; Bondi et al., 2010). These factors allow fibroblasts to assist in the activation and migration of resident immune cells such as macrophages. Moreover, the recruitment of non-resident immune cells is facilitated by the fibroblast-mediated production and maintenance of the relatively spacious, non-solid ground substance of the extracellular matrix, which plays an important role as a thoroughfare for the extravasation of immune cells into connective tissue. These tools endow fibroblast roles in chemical (non-specific) and cell-mediated immunity, acute and chronic inflammation, and inflammation resolution. Fibroblasts can contribute to chronic inflammation (Flavell et al., 2008), and reciprocally, inflammatory cytokines promote fibroblast to myofibroblast transition, facilitating fibrosis.

### Angiogenesis

Fibroblasts have close interactions with endothelial cells and facilitate angiogenesis into tissues beyond the reach of existing blood vessels. This response requires the migration of endothelial cells to construct tubes through the ground substance of connective tissue (Dunn et al., 2000). Fibroblasts play a critical role in angiogenesis; fibroblast-derived matrix proteins and cytokines are essential for endothelial cell-mediated lumen formation (Newman et al., 2011). A major mechanism for this phenomenon is the fibroblast-mediated production and release of vascular endothelial growth factor (VEGF), which acts on VEGF receptors expressed on endothelial cells to promote angiogenesis. Interestingly, dermal fibroblasts from SSc patients overexpress VEGF in response to autocrine TGFβ signaling (Kajihara et al., 2013). This VEGF could play a role in the vascular damage that in turn promotes fibroblast activation, thereby supporting fibrosis (Kajihara et al., 2013).

## Ancillary signaling functions of fibroblasts

**Figure 1:**
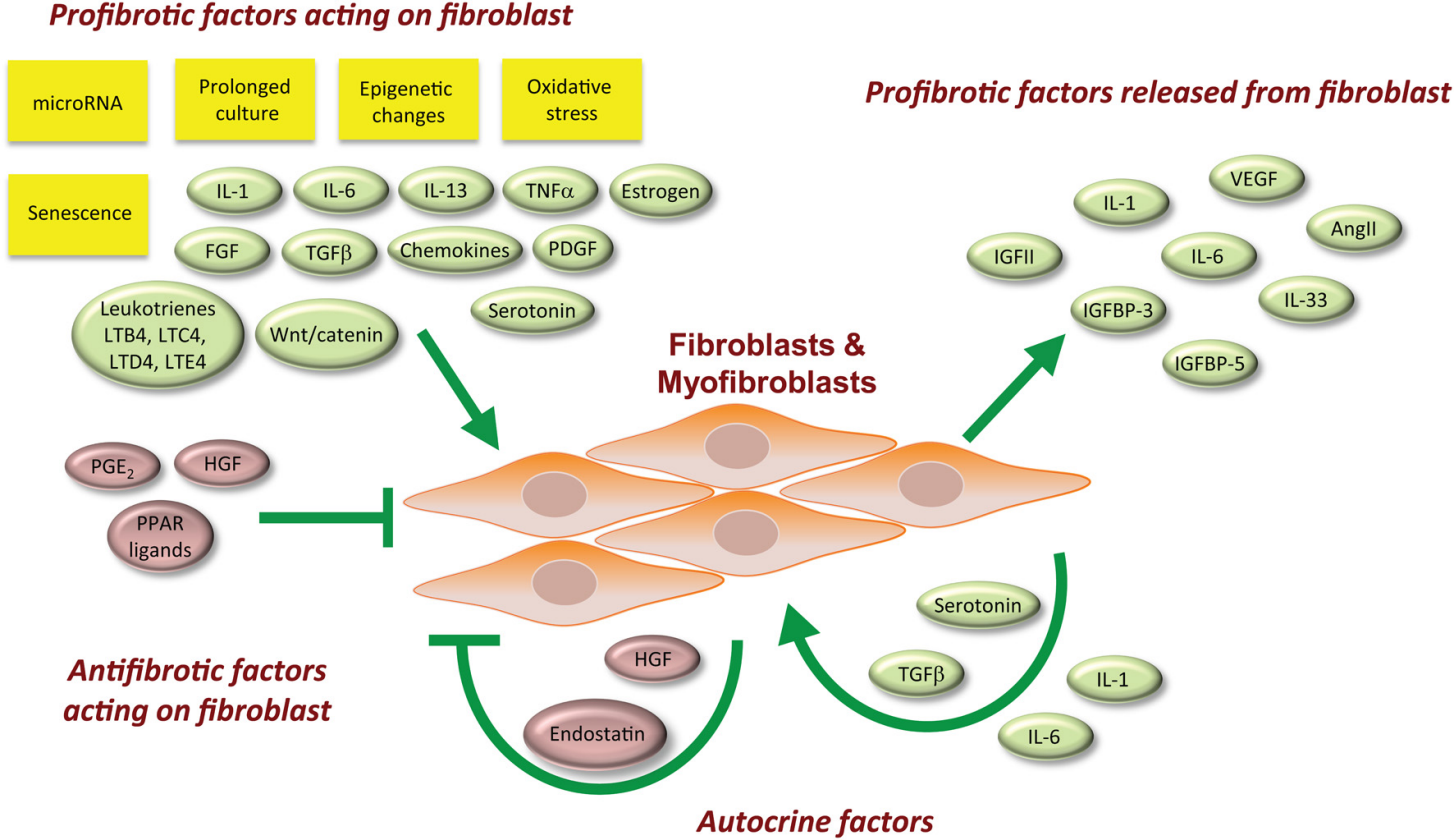
**Overview of factors involved with the promotion of a profibrotic myofibroblast phenotype**.

The signaling factors associated with fibroblast biology are not necessarily specific for one biological role over another. Below we discuss these signaling factors with respect to their sources and their cellular targets (Figure 1). The net effect of multiple stimuli that recapitulate the milieu in human tissues is difficult to reproduce in the laboratory, although initial efforts using *ex vivo* organ cultures such as skin (Yasuoka et al., 2008; Yamaguchi et al., 2012) are promising.

### Paracrine signals that act on fibroblasts

Some factors act to promote a profibrotic phenotype. Platelet-derived growth factor (PDGF) (Scotton and Chambers, 2007), IL-6 (Feghali et al., 1992), IL-13 (Fuschiotti, 2011; Fuschiotti et al., 2013), and the eicosanoid leukotrienes (Mensing and Czarnetzki, 1984; Chibana et al., 2003; Eap et al., 2012) are cytokines that promote inflammation and the development of a fibrotic response. PDGF is produced by a variety of cell types, such as platelets, endothelial cells, smooth muscle cells, and macrophages. It acts on the mesenchyme and fibroblasts to induce proliferation, differentiation, and ECM production (Scotton and Chambers, 2007). PDGF is important for wound healing, inflammation, angiogenesis, embryonic development, and fibrosis. IL-6 is a pro-inflammatory cytokine that acts on a range of cell types and is produced by T cells, macrophages, skeletal muscle cells, and fibroblasts (Feghali and Wright, 1997). IL-6 has well-known roles in inflammation, cellular differentiation, and fibrosis. IL-6 is elevated in the blood of SSc patients and produced in excess by SSc fibroblasts (Feghali et al., 1992; Feghali and Wright, 1997). Elevated IL-6 levels correlate with worse long-term survival, and therefore IL-6 could potentially be used as a prognostic clinical biomarker for patients with diseases such as SSc (Khan et al., 2012). IL-13 is a cytokine mainly produced by mast cells but also T lymphocytes (Wynn, 2003), and it acts on multiple cell types, including fibroblasts. With roles in both inflammation and fibrosis, IL-13 can stimulate TGFβ production, TGFβ activation, collagen production, MMP expression, fibroblast proliferation, and myofibroblast differentiation (Fuschiotti, 2011; Fuschiotti et al., 2013). The leukotriene eicosanoids are additional signals that induce fibroblast proliferation and matrix production contributing to fibrosis (Mensing and Czarnetzki, 1984; Chibana et al., 2003; Eap et al., 2012).

Of special note, the fibroblast growth factor (FGF) family of genetically related protein ligands was originally identified as mitogens and differentiation factors for fibroblasts (Armelin, 1973; Gospodarowicz, 1974). Despite the fact that this family has since been found to act on multiple cell types, FGFs require heparin or heparan sulfate proteoglycan molecules to bind their surface receptors (Ornitz and Itoh, 2001). Therefore, they require close proximity to ECM and the fibroblasts that secrete ECM. There are 22 FGF genes found in humans (Ornitz and Itoh, 2001), and these genes are alternatively-spliced (Ornitz and Itoh, 2001) generating additional FGF proteins. FGF ligands activate a family of 4 FGF receptors with differing specificities (Dionne et al., 1991). High amounts of active FGF2 are found in bronchoalveolar lavage fluid and in lung tissues of patients with pulmonary fibrosis (Henke et al., 1991; Inoue et al., 1996). Mast cell-generated FGF2 correlates with the localization and the extent of fibrosis in IPF (Inoue et al., 1996, 2002).

Paracrine factors that can antagonize fibrosis have also been described. The eicosanoids prostaglandin E2 (PGE_2_) (Keerthisingam et al., 2001; Maher et al., 2010) and prostacyclin (PGI_2_) (Stratton et al., 2002) are two examples of paracrine signals that inhibit the fibrotic response. PGE_2_ inhibits fibroblast proliferation and collagen production (McAnulty et al., 1997; Goldstein and Polgar, 1982; Saltzman et al., 1982). It acts on target cells by activating the G protein-coupled EP receptors 1–4. PGE_2_ has been observed to inhibit fibroblast to myofibroblast differentiation and the TGFβ1 stimulated expression of α smooth muscle actin (αSMA) in lung fibroblasts via the EP2 subtype receptors (Kolodsick et al., 2003). PGE_2_ also has an anti-apoptotic effect on the alveolar epithelium (Maher et al., 2010). Iloprost is a synthetic analog of prostacyclin eicosanoid PGI_2_ that dilates systemic and pulmonary arterial vascular beds and is used for the treatment of patients with SSc (Erre and Passiu, 2009).

### Paracrine signals produced and broadcast by fibroblasts

IGFBP-3 and -5, IGF-II, connective tissue growth factor (CTGF), IL-33, CXC chemokines, CC chemokines, and reactive oxygen species (ROS) are paracrine signals produced by fibroblasts that further enhance a tissue fibrotic response. Both IGFBP-3 and -5 are profibrotic factors implicated in SSc and IPF (Veraldi et al., 2009; Veraldi and Feghali-Bostwick, 2012). CTGF plays pivotal roles in wound healing, angiogenesis, and fibrosis. It is highly expressed by endothelial cells in response to a variety of stressors, but it is also expressed and released by fibroblasts. CTGF interacts with a wide variety of receptors, extracellular ligands, as well as ECM proteins (Liu et al., 2011a). CTGF knock-out mice are less responsive to the induction of fibrotic skin lesions by bleomycin (Liu et al., 2011a). Conversely, transgenic mice with fibroblast-specific expression of CTGF develop fibrosis without any other profibrotic stimulus and display an accelerated fibrotic response relative to exogenously added CTGF (Sonnylal et al., 2010). CTGF is well-characterized to act synergistically with TGFβ to promote pathological fibrosis. Furthermore, TGFβ1 induces CTGF in fibroblasts (Leof et al., 1986; Paulsson et al., 1987; Soma and Grotendorst, 1989; Igarashi et al., 1993; Mori et al., 1999). IL-33 is a cytokine produced primarily by endothelial and smooth muscle cells in the vasculature and the lungs. However, its production by dermal fibroblasts can also be induced by inflammation and in cardiac fibroblasts by mechanical stress. Knocking down IL-33 expression in mice is cardioprotective in the face of chronic angiotensin II stimulation (Sanada et al., 2007). IL-33 facilitates inflammation and fibrosis (Savinko et al., 2012). CXC and CC chemokines are broadcast by fibroblasts and play significant roles in the chemotaxis of various leukocytes to sites of tissue injury (Gharaee-Kermani et al., 2003, 2012). TGFβ also induces NADPH oxidase 4 (NOX4) in fibroblasts (Amara et al., 2010; Bondi et al., 2010), implicating reactive oxygen species (ROS) and oxidative stress in the pathology of fibrosis. NOX4 generates a basal level of extracellular H_2_O_2_. NOX4 mRNA expression is increased in rat kidney fibroblasts in culture (Bondi et al., 2010) and in primary lung fibroblasts derived from the lungs of patients with IPF (Amara et al., 2010).

Fibroblasts do not typically express and release the angiogenic hormone angiotensin II (AngII), however both activated macrophages and myofibroblasts produce AngII during the pathology of fibrosis (Berk et al., 2007). Although the phenomenon is not well characterized in most tissues, AngII is known to promote TGFβ-mediated cardiac remodeling (Rosenkranz, 2004) and the fibrosis observed in left ventricular cardiac hypertrophy (Rosenkranz, 2004). These findings suggest that AngII could contribute to fibrosis in other tissues.

### Autocrine actions among fibroblasts

TGFβ (Scotton and Chambers, 2007), IL-1β, and IL-6 (Feghali and Wright, 1997) are examples of cytokines that are both produced by fibroblasts (as well as other cell types) and that act on fibroblasts to promote inflammatory and fibrotic responses. TGFβs are named after their ability to act on fibroblasts to induce oncogenic transformation. The three known TGFβ ligands assert their biological actions by activating TGFβ receptors, cell surface serine/threonine kinase receptors. TGFβ is the prototypic profibrotic cytokine. It acts on fibroblasts and myofibroblasts to promote proliferation, migration, matrix production, the production of chemotactic signals promoting leukocyte recruitment to the site of injury, fibrosis, and the differentiation of fibroblasts to myofibroblasts. Inhibitors of each TGFβ receptor antagonize fibrosis development in experimental models (Bonniaud et al., 2005; Fu et al., 2011). The cytokine IL-1β's expression and release is induced by a wide variety of cells, including fibroblasts, in response to inflammation (Feghali and Wright, 1997). IL-1β is a potent pro-inflammatory cytokine that also induces production of the profibrotic cytokines PDGF and TGFβ.

Some autocrine fibroblast factors counteract fibrosis, such as the hepatocyte growth factor (HGF). HGF is a protein produced by fibroblasts and acts by binding the receptor tyrosine kinase c-Met expressed on nearby epithelial cells, endothelial cells, and fibroblasts (Bogatkevich et al., 2007a,b; Crestani et al., 2012). HGF also plays an important role in wound healing, angiogenesis, and tumorigenesis.

## Sources of fibroblasts

Fibroblasts are a heterogeneous cell population that consists of subsets with different capacities to produce ECM components such as collagen. Fibroblast subpopulations have been described in the skin (Jelaska and Korn, 2000) and lung (Pechkovsky et al., 2010). Furthermore, fibroblast phenotypes may differ across sites of the same organ, as has been shown in skin. In fact, fibroblasts from different skin sites retain positional memory with distinct gene expression profiles (Chang et al., 2002). Furthermore, fibroblasts are chemotactic and can migrate and accumulate in new areas in response to secreted cytokines, a behavior well characterized in the wound healing response after tissue injury. Although the parenchyme of the liver, pancreas, and lung are derived from endothelial tissue and the parenchyme of the skin and brain are derived from epithelial tissue, the stromal connective tissue found in all organs is derived from mesothelial tissue. Yet, due to the differing mixture of neighboring tissue types, there is the potential for differential fibroblast phenotypes and differential fibrotic responses in each of these organs.

**Figure 2:**
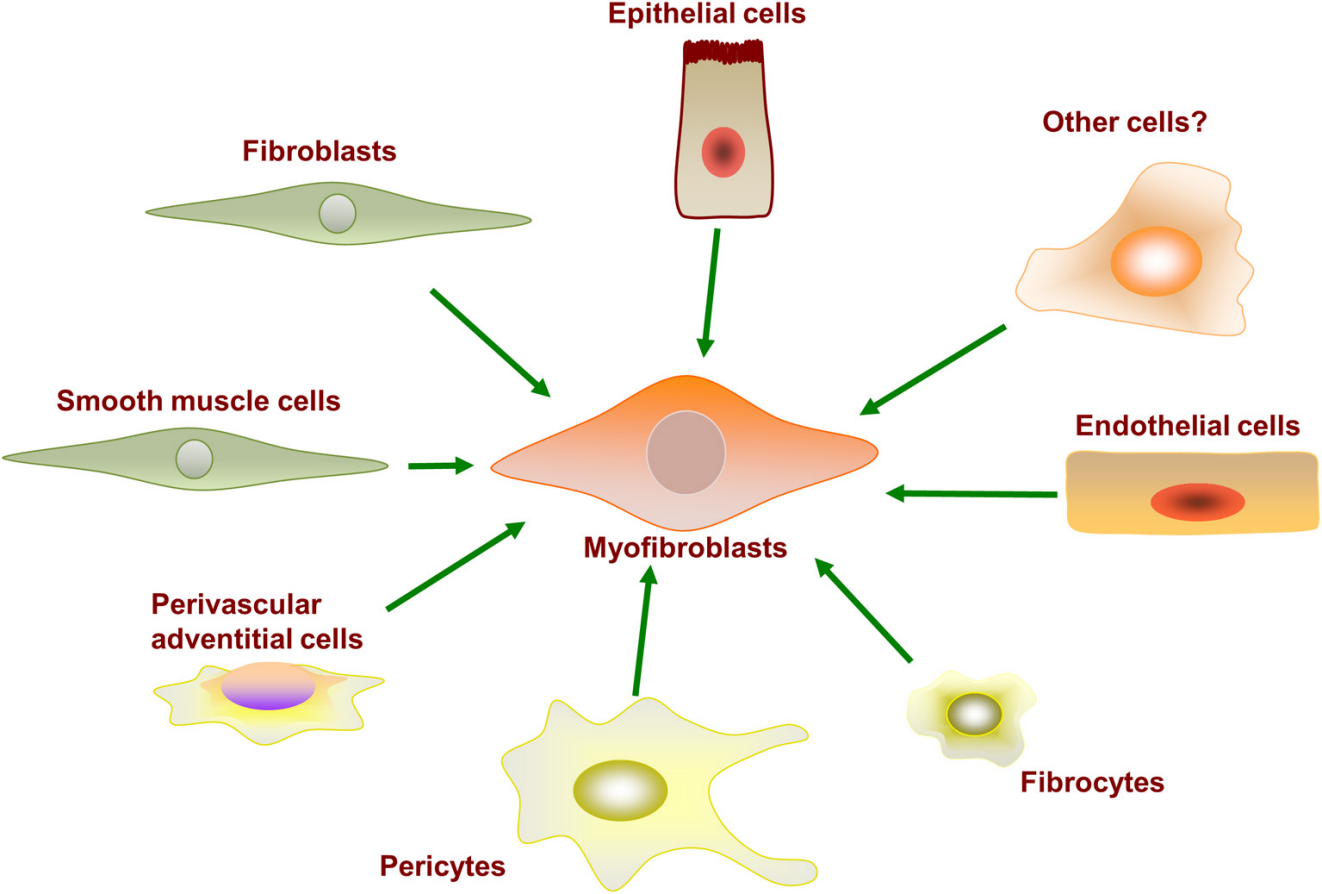
**Myofibroblasts can differentiate from a variety of precursor cell types**.

Fibroblasts are not a terminally differentiated cell type and retain the potential to be activated for differentiation into subtypes of fibroblast-like cells. Myofibroblasts are rarely found in healthy human physiology; they become vastly up-regulated after injury and play a critical role in the wound healing response (Gabbiani, 2003; Midwood et al., 2004). Myofibroblasts can originate from a variety of precursor cells, as shown in Figure 2. Major developmental signaling factors such as the Wnt (Liu et al., 2012), Notch (Kavian et al., 2012), and Sonic hedgehog ligands (Stewart et al., 2003; Ding et al., 2012) have well-described roles in myofibroblast differentiation from precursor cells. Myofibroblasts are well characterized to differentiate from resident fibroblasts *in vivo* and *in vitro* in response to profibrotic cytokine stimulation. However, myofibroblasts have also been observed to differentiate from various other precursor cells, including epithelial cells, endothelial cells, pericytes, multipotent monocytes, and fibrocytes. Whether different cell origins are the source of myofibroblasts in different tissues remains to be determined.

The myofibroblast phenotype is characterized as: (i) expressing αSMA; (ii) contractile; (iii) having a greater rate of ECM synthesis and secretion; and (iv) resistant to apoptosis. The contractile biology of myofibroblasts facilitates ECM fiber manipulation and wound contraction at sites of injury. Myofibroblasts display a heightened and constitutive expression of cytokines, chemokines, and cell surface receptors (Strehlow and Korn, 1998; Abraham and Varga, 2005). These differentiated myofibroblasts also display epigenetic changes (Wang et al., 2006a).

### Epithelial cells

The EMT has been observed as a source of myofibroblasts in kidney fibrosis (Ng et al., 1998; Jinde et al., 2001; Liu, 2004); however, subsequent attempts to trace such phenomena in the skin, lung, heart, and liver have been largely unsuccessful *in vivo*, suggesting that EMT-mediated formation of myofibroblasts is not substantial. Recent evidence argues against epithelial cells as the main source of myofibroblast differentiation in idiopathic lung fibrosis. Using lineage tracing with recombinant td-Tomato and f-GFP tags and confocal microscopy resolution, Rock et al. demonstrated focal accumulation of fibroblasts derived from the proliferation of stromal cells (Rock et al., 2011). Recent transgenic lineage tracing studies in mouse kidney also indicate that most myofibroblasts are not derived from epithelial cells (Lin et al., 2008; Humphreys et al., 2010). Although these studies using rodents contradict findings suggesting an epithelial origin to myofibroblasts, they do not exclude the possibility that EMT may occur in human tissues.

### Endothelial cells

Another source of myofibroblasts during fibrosis has been hypothesized to originate from endothelial cells by a process of endothelial-mesenchymal transition (EndoMT) (Piera-Velazquez et al., 2011). This event has been characterized in animal models of fibrosis; however, similar phenomena in human fibrosis patients have not yet been well characterized.

### Vascular smooth muscle cells (VSMC)

The many similarities between myofibroblasts and VSMCs promote the question of a potential lineage relationship between the two cell types (Yoshida and Owens, 2005). For example, these cells are contractile and they share multiple markers such as αSMA, SM22α, desmin, and vimentin (Yoshida and Owens, 2005). Furthermore, myofibroblasts express proteins, such as osteopontin, that can be induced in VSMCs by phenotypic modulator ligands (Yoshida and Owens, 2005). However, the biological roles of these two cell types in disease are different (Gan et al., 2007). Furthermore, myofibroblasts and VSMCs have distinct transcriptional mechanisms for αSMA expression (Gan et al., 2007). Observations such as the induction of αSMA expression in myofibroblasts and the reduction in αSMA expression in VSMCs during vascular injury (Yoshida and Owens, 2005) suggest the reduction of a contractile phenotype in VSMCs could also promote another source of myofibroblasts differentiation.

### Pericytes

Pericytes are mesenchyme-derived cells that wrap around small blood vessel walls and closely associate with the outer surface of the endothelial cell layer. Pericytes regulate the permeability of the blood vessel wall; for example, pericytes are the principal cell type that regulates the permeability of the blood–brain barrier (Armulik et al., 2010). Pericytes physically interact with endothelial cells and chemically via paracrine signaling to control the tight junctions and transvessicular trafficking across endothelial cells. As such, malfunctioning pericytes can promote vascular leaking (Schrimpf et al., 2012), and the observation that perictyes can be induced to differentiate into myofibroblasts, losing their pericyte functionality, suggests that this differentiation may contribute to both tissue fibrosis and vascular leakage in IPF (Hung et al., 2013). Pericytes are not a terminally differentiated cell type; they can further differentiate into fibroblasts, osteoblasts, and smooth muscle cells. Pericytes endogenously express αSMA and have a functioning contractile apparatus; therefore, distinguishing between these cells and other cell types is challenging. One genetic fate mapping study investigating the potential contribution of pericytes in mice to bleomycin-induced lung fibrosis utilized the nerve/glial antigen 2 (NG2) protein, a chondroitin sulfate cell surface-associated proteoglycan, as a marker for this cell type. This strategy found no appreciable contribution of pericytes to the expansion of myofibroblasts and the resulting fibrosis even though the pericyte population itself did expand significantly (Rock et al., 2011). A different investigation utilized the forkhead transcription factor Foxd1 as a marker for lung pericytes and found that up to 68% of αSMA-expressing cells in fibrotic lungs are pericyte-derived (Hung et al., 2013). These studies suggest that multiple subpopulations of pericytes may exist in the lung and differentially contribute to myofibroblast formation and fibrosis.

### Monocytes and fibrocytes

Fibrocytes are circulating fibroblast-like cells in the vascular system that are derived from bone marrow stem cells (Bucala et al., 1994). “Fibrocyte” is a term sometimes ascribed to a relatively inactive fibroblast-like cell, whereas the term “fibroblast” designates a fully active cell as described throughout our review. Fibrocytes can extravasate from vessels into connective tissues. In the kidney, it has been proposed that myofibroblasts in fibrotic areas could be derived from bone marrow progenitor multipotent cells via a cellular differentiation lineage: bone marrow progenitor cell to monocyte to fibrocyte to fibroblast (Quan et al., 2004; Pilling and Gomer, 2012). Circulating CD14^+^ immunophenotypic monocytes derived from the plasma of SSc patients are reported to express greater αSMA (and so an increased maturation toward myofibroblasts) compared to those of healthy control individuals but with minor functional contraction properties as determined with a collagen contraction assay (Binai et al., 2012). The blood of SSc patients with interstitial lung disease is enriched for fibrocytes and monocytes with an overt profibrotic phenotype (Mathai et al., 2010). However, lineage tracing studies in rodents display differing conclusions. GFP-expressing fibroblasts derived from the multipotent, transgenic bone marrow transplanted into chimeric normal mice appear to be a unique population of cells that do not express αSMA and fail to differentiate into myofibroblasts when extracted and stimulated *ex vivo* in culture (Hashimoto et al., 2004; Kisseleva et al., 2006; Barisic-Dujmovic et al., 2010; Rock et al., 2011). Similarly, using an αSMA promoter-driven GFP transgenic mouse, no kidney or bone marrow stromal expression was observed in chimeric wild-type mice (Yokota et al., 2006). However, the chimeric mice were only analyzed under physiologic conditions, and no profibrotic stimulus was tested in this model (Yokota et al., 2006). In contrast, in another study, bone marrow from GFP transgenic mice were transplanted into wild-type rats, and a 23% increase in the number of GFP and αSMA-expressing cells in the pancreas was observed to peak in the early stage of an experimentally-induced but clinically-relevant pancreatitis rat model (Mathai et al., 2010; Akita et al., 2012). In all, despite substantial evidence that activated myofibroblasts are derived from local stromal fibroblasts, blood borne monocytes and monocyte-derived cells appear to facilitate fibrosis via the production of ECM and paracrine signaling with resident profibrotic cells (Murray et al., 2012).

## Adventitial fibroblasts in pulmonary hypertension

A very similar role of fibroblast activation appears to promote pulmonary hypertension compounding other fibrotic disorders. Pulmonary hypertension can be a complication of IPF and SSc and is a significant contributor to mortality (Lee et al., 1992; Arcasoy et al., 2001; King et al., 2001). Adventitial fibroblasts found in blood vessels can be activated by hypoxia and are implicated as a source of myofibroblasts that promote vascular remodeling and the associated pulmonary hypertension (Stenmark et al., 2006).

## Fibroblasts in cancer biology

Fibroblasts have an integral role in cancer beyond sarcomas and cancers originating from fibroblastic cells. Non-cancerous fibroblasts interact with cancer cells, affecting tumor biology and pathogenesis. The biological processes of wound healing, inflammation, fibrosis, angiogenesis, and their associated biochemical machinery all play critical roles in cancer by regulating the microenvironment of a tumor, tumor size, and tumor invasion of adjacent tissues and metastasis (Mueller and Fusenig, 2004). Fibroblasts in and around tumors are persistently activated by tumor cells (Kalluri and Zeisberg, 2006). In response, fibroblasts secrete cytokines and ECM that modulate tumor progression and regulate stroma-cancer interactions (Madar et al., 2013). Non-cancerous fibroblasts surrounding tumors contribute directly to angiogenesis and tumor growth by producing multifarious signaling molecules (Madar et al., 2013). Fibroblasts also secrete the ECM that modulates tumor progression, and fibroblasts remodel the connective tissue surrounding a tumor, allowing cancerous cells to be released from a contained tumor into the vascular system, thereby promoting metastasis (Liu et al., 2011b). As such, fibroblast cells also participate in the pathogenesis of carcinomas—the more abundant cancers originating from epithelial cells. In spite of recent advances in delineating the role of fibroblasts in tumor progression, the role of these cells in earlier tumor development is still incompletely understood.

## Potential therapies targeting fibroblasts

Since fibroblasts are a central effector cell in fibrosis, they are suitable therapeutic targets. There have been some recent exciting research findings inciting potential strategies to combat the pathological fibrotic response that occurs in various disorders. The examples listed below are not intended to be a comprehensive presentation for the pipeline of future antifibrotic therapies that target the fibroblast cell lineage. There are multiple strategies being investigated that are too numerous to cover here. The following examples help to illustrate the diverse range of targeting strategies where novel therapies potentially could ensue.

### Endostatin

Endostatin is an endogenous inhibitor of angiogenesis that has completed clinical trials as an anticancer agent to prevent the growth of tumor mass. It inhibits endothelial cell proliferation and vessel formation (O'Reilly et al., 1997) but also acts on fibroblasts (Yamaguchi et al., 2012). Endostatin is a small peptide (20–30 KDa) derived from the carboxy terminus of the collagen type XVIII produced and released by fibroblasts. Endostatin is then released from this precursor protein by proteolytic cleavage by the action of cathepsin L (Felbor et al., 2000), and peptides of differing lengths have also been reported to be generated by the matrix metalloproteases MMP-3, -7, -9, -13, or -20 (Heljasvaara et al., 2005). Endostatin concentrations are greater in the blood and lungs of patients with IPF and SSc, but these concentrations do not seem to reach “therapeutic” levels (Hebbar et al., 2000; Sumi et al., 2005; Richter et al., 2009). Interestingly, the amino terminus portion of endostatin contains the anti-angiogenic functionality (Tjin Tham Sjin et al., 2005), whereas the carboxy terminus portion contains the antifibrotic activity, suggesting that the two biological functions can be attributed to dissociable domains (Yamaguchi et al., 2012). Indeed, a 48 amino acid portion of endostatin's carboxy terminus has strong antifibrotic actions *in vitro*, *in vivo*, and *ex vivo* in human tissue (Yamaguchi et al., 2012). This peptide ameliorates pulmonary and dermal fibrosis in an animal model and *ex vivo* in human skin in response to TGFβ (Yamaguchi et al., 2012).

### Vitamin D3

Vitamin D is important for regulating mineral absorption in the intestines, calcium and phosphate concentrations in blood, and for maintaining bone integrity. However, vitamin D deficiency has also been found to correlate with many extra-osseous disorders. Vitamin D3 is processed by the liver to form the biologically active form 1,25-dihydroxyvitamin D3 (1,25-(OH)_2_ vitamin D3). 1,25-(OH)_2_ vitamin D3 activates the vitamin D receptor (VDR, a steroid hormone receptor) that heterodimerizes with retinoid hormone receptor to directly regulate gene transcription. 1,25-(OH)_2_ vitamin D3 inhibits the expression of type I collagen in hepatic stellate cells (Potter et al., 2013). Furthermore, 1,25-(OH)_2_ vitamin D3 has been observed to inhibit a fibrotic response in human primary lung fibroblasts (Ramirez et al., 2010), and 1,25-(OH)_2_ vitamin D3 deficiency correlates with the severity of liver fibrosis and that of SSc (Vacca et al., 2009; Caramaschi et al., 2010; Rios Fernandez et al., 2010; Arnson et al., 2011; Slominski et al., 2013). These studies suggest that the correction of vitamin D deficiency is promising as a clinical strategy to alleviate fibrotic disorders.

### Epigenetics

Epigenetic changes in fibrosis are the subject of active investigation. Several epigenetic modifications, like histone posttranslational modifications and DNA methylation events, have been reported. The fact that these epigenetic modifications do not alter the DNA sequence suggests they are reversible, and as such, they represent an attractive therapeutic strategy for countering fibrosis. Cyclooxygenase 2 (COX2) expression—a biosynthetic enzyme for antifibrotic prostaglandin production—is suppressed in IPF due to decreased histone acetylation (Coward et al., 2009), and histone deacetylase 4 (HDAC4) activity mediates TGFβ stimulated myofibroblast differentiation in a response that can be inhibited by global inhibition of HDACs (Glenisson et al., 2007). HDAC7 has been proposed as a potential target for the treatment of SSc (Hemmatazad et al., 2009). DNA methylation of the gene FLI1 coding for the ETS transcription factor Fli-1 inhibits its genetic expression, resulting in enhanced collagen type 1 expression and persistent activation of fibroblasts in SSc (Wang et al., 2006a). Primary fibroblasts isolated from the adventitial layer of blood vessels following vascular injury maintain their activated, pro-fibrotic state when grown *in vitro* (Li et al., 2011). The constitutive activation is an imprinted phenotype maintained by epigenetic alterations in key inflammatory and pro-fibrotic genes (Li et al., 2011). Epigenetic drugs have been approved for cancer, paving the way for using such agents, including DNA demethylating agents, for other diseases. The reversibility of epigenetic alterations and the ready availability of small molecule inhibitors of these enzymes provide a potential future therapeutic strategy that could antagonize the profibrotic phenotype of fibroblasts.

### Wnt-like signal transduction pathway

Wnt signaling and a classical Wnt-like intracellular signal transduction pathway in fibrosis are a bustling area of research, and multiple proteins within this pathway have been proposed as druggable targets to prevent myofibroblast differentiation and fibrosis. Wnt is a paradigm developmental signaling protein that regulates proliferation, migration, and cell fate determination. Wnt ligands activate frizzled receptors—members of the G protein-coupled receptor (GPCR) superfamily of proteins that share common structure and function relationships—and promote the inhibition of glycogen synthase kinase 3β (GSK3β). Interestingly, many other GPCRs such as the lysophosphatidic acid receptor, the angiotensin type I receptor, the endothelin 1 receptor, and CC and CXC chemokine receptors regulate myofibroblast differentiation and fibrosis. GPCRs have also been demonstrated to couple to the Wnt-like intracellular signal transduction cascade Akt-GSK3β-βcatenin-PPARγ (Beaulieu et al., 2004, 2005, 2008; Kendall et al., 2011). However, it is not yet known if this cascade is the main contributor for GPCR-regulation of myofibroblast transition and fibrosis. Regardless, it is an interesting observation that multiple targets belonging to this signaling pathway are currently being actively investigated as potential targets for fibrosis therapies.

There is an apparent activation of the developmental Wnt-βcatenin program in SSc (Bhattacharyya et al., 2012). Wnt activated βcatenin promotes fibroblast to myofibroblast transition in human skin (Liu et al., 2012) and is required for TGFβ-mediated fibrosis (Akhmetshina et al., 2012). While Akt is not required for Wnt-stimulated catenin accumulation (Torres et al., 1999; Chen et al., 2000), Akt has been implicated in sustaining the Wnt signaling cascade (Fukumoto et al., 2001). Akt is a critical node of PI3K signaling and regulates cell cycle, cell growth, and cell fate determination. PI3K-Akt signaling is also utilized as a non-canonical pathway of TGF receptors that contribute to fibrosis (Wan et al., 2013). Sustained Akt activation is critical for upregulating αSMA expression and myofibroblast differentiation in the wound healing response, implicating Akt as a potential therapeutic target for fibrosis. Whereas a constitutively active Akt1 protein (myr-Akt1) induces αSMA expression in fibroblasts, a dominant negative Akt1 (Akt1 K179M) inhibits αSMA expression (Abdalla et al., 2013). Akt is a ubiquitous kinase that phosphorylates and inactivates the constitutive activity of GSK3β (Fang et al., 2000). Knocking out GSK3β expression in fibroblasts accelerates wound healing, increases fibrosis, enhances collagen production, decreases cell apoptosis in wound sections, increases profibrotic αSMA expression, and increases myofibroblast formation (Kapoor et al., 2008). The transcriptional coregulator βcatenin is required for the TGFβ-mediated inhibition of peroxisome proliferator-activated receptor γ (PPARγ) expression in hepatic stellate cells (Qian et al., 2012). PPARγ is a nuclear receptor that is important for mesodermal cell fate determination. PPARγ expression and activity promotes adipocyte differentiation and inhibits fibroblast and hepatic stellate cell activation of the profibrotic phenotype (Qian et al., 2012). PPARγ agonists inhibit lung myofibroblast proliferation *in vivo* as well as TGFβ-mediated myofibroblast differentiation and collagen production in culture (Milam et al., 2008). PPARγ agonists also inhibit dermal fibrosis (Wei et al., 2010, 2012). Although only an associative depiction of a pathway is characterized here from research on individual proteins in this pathway, collectively they suggest that a Wnt-like pathway could have a strong influence on myofibroblast differentiation and fibrosis.

### Fibroblast receptors

Caveolin 1 inhibits the function of the chemotactic receptor CXCR4 in fibrocytes (Tourkina et al., 2011) and caveolin 1 is down regulated in IPF (Wang et al., 2006b). The introduction of a peptide containing the caveolin scaffolding domain of caveolin 1 is sufficient to inhibit fibrocyte accumulation in the lungs in a bleomycin-induced animal model of fibrosis, suggesting that the caveolin scaffolding domain could be employed as a potential therapy for SSc lung disease (Tourkina et al., 2011). In yet another example, the single nucleotide polymorphism L412F in the gene expressing Toll-like receptor 3 in pulmonary fibroblasts has been found to correlate with a greater risk of mortality in IPF patients (O'Dwyer et al., 2013). The L412F polymorphism could be used as a biomarker for more progressive fibrotic disease, and the resulting loss of function of TLR3 is a potential therapeutic target in IPF (O'Dwyer et al., 2013). This study highlights the importance of a personalized medicine approach to the multifactorial group of fibrotic disorders. The inhibition of TLR4 has been reported to antagonize AngII-induced cardiac fibrosis (Wang et al., 2014), the activity of TLR4 in hepatic stellate cells enhances liver fibrosis (Seki et al., 2007), and the activity of TLR4 in skin fibroblasts enhances SSc (Bhattacharyya et al., 2013). Here, the entanglement of inflammatory and ECM-producing functions of fibroblasts can be exploited to improve fibrotic therapies (Bhattacharyya et al., 2013).

Given the multifactorial etiology of various fibrotic disorders, both in different tissues and within the same tissue (Murray et al., 2012), the increasing interest in developing and employing personalized medicine, where a one-size-fits-all approach is unlikely to be as effective, offers to accelerate the development of more effective antifibrotic therapies for defined subsets of patients.

## Summary

Fibrosis is the thickening of ECM that is preceded by inflammation or physical tissue injury. Fibroblasts are the principal cell type that produces, maintains, and reabsorbs ECM. These fibroblasts have the capacity to become activated by inflammatory cytokines to myofibroblasts that display up-regulated cellular migration, exaggerated ECM production, the endowment of a contractile apparatus, and increased chemical signaling secretion and responsiveness. Although various precursor cell types have been observed to contribute to myofibroblast development, resident fibroblast stromal cells appear to be the most significant contributing source of myofibroblasts and the resulting tissue fibrotic response in diseases such as SSc and IPF. Thus, fibroblasts are a suitable therapeutic target. In view of the numerous factors at play in fibrosis, it is likely that combinatorial therapy that includes targeting fibroblasts will be more effective than single target approaches for the treatment of organ fibrosis.

### Conflict of interest statement

The authors declare that the research was conducted in the absence of any commercial or financial relationships that could be construed as a potential conflict of interest.
